# The Global Experiment: How the International Atomic Energy Agency Proved Dosimetry to Be a Techno-Diplomatic Issue

**DOI:** 10.1007/s00048-022-00336-9

**Published:** 2022-05-10

**Authors:** Maria Rentetzi

**Affiliations:** 1 Friedrich-Alexander-Universität Erlangen-Nürnberg, Erlangen, Germany; 2 Max Planck Institute for the History of Science, Berlin, Germany

**Keywords:** Radiation dosimetry, Radiation protection, Radiation therapy, International Atomic Energy Agency, Nuclear history

## Abstract

This paper draws attention to the role of the IAEA in shaping radiation dosimetry practices, instrumentation, and standards in the late 1950s and 1960s. It traces the beginnings of the IAEA’s radiation dose intercomparison program which targeted all member states and involved the WHO so as to standardize dosimetry on a global level. To standardize dosimetric measurement methods, techniques, and instruments, however, one had to devise a method of comparing absorbed dose measurements in one laboratory with those performed in others with a high degree of accuracy. In 1964 the IAEA thus started to build up what I call the “global experiment,” an intercomparison of radiation doses with participating laboratories from many of its member states. To carry out the process of worldwide standardization in radiation dosimetry, I argue, an organization with the diplomatic power and global reach of the IAEA was absolutely necessary. Thus, “global experiment” indicates a novel understanding of the experimental process. What counts as an experiment became governed by a process that was designed and strictly regulated by an international organization; it took place simultaneously in several laboratories across the globe, while experimental data became centrally owned and alienated from those that produced it.

“Many radioisotope teletherapy units are now in use in establishments where neither the staff nor the facilities required to make complete dosimetric measurements are available” reported the International Atomic Energy Agency in 1961.^1^ Until then in medical centers throughout the world radiotherapy, also known as radiation therapy—the use of high-energy radiation to kill cancer cells and thus shrink tumors—was carried out mainly by means of X‑ray machines and radium applicators. During the pre-atomic era, X‑ray therapeutic equipment emitted radiation of low energy and limited penetrating power, operating at 250 to 400 kv; it was difficult to maneuver and radiation doses were hard to estimate. X‑ray tubes were placed close to the skin with little protection (Mould 1993; Womack 2020). In addition, the use of needles, tubes, and other crude radium applicators for the treatment of cancer often proved to be hazardous for both the patients and their caregivers. Weighed against their side effects, these methods were ineffective in the treatment of especially deep-seated cancers (Lederman 1981; Rentetzi 2022).

Throughout the 1950s, major medical centers in advanced countries switched to other, more powerful sources of radiation. Many of these were widely accepted and used, such as the new isotopic teletherapy units, which contained radioactive isotopes such as cobalt-60, and X‑ray machines in the so-called supervoltage range; both of these technologies generated high-energy electron beams. These sources facilitated the work of physicians, allowing for better estimation and localization of radiation doses to the targeted cancerous tissues (Boone et al. 1977).

Gradually, the betatron and the cyclotron were introduced into cancer research and took the place of the electrostatic generators that produced X‑rays. And while advanced countries like the United States, Canada, Sweden, and Britain were the first to adopt such sophisticated medical technologies, these delicate machines, which required experience in their handling, skilled personnel, and a great deal of supervision, were also used in less technologically advanced parts of the world.

This rapid development in the adoption of new medical technologies created new concerns. Radiation dosimetry in medicine became an issue of high priority for both the patient and the personnel involved in administering the treatment (Mould 1993). The early dosimetric methods of the 1920s and 1930s had been based on the chemical effects of ionizing radiation. During the 1940s photographic dosimetry and the use of film badges were introduced in radiation facilities both medical and industrial. Developments in chemical instrumentation and theories during the 1950s led to the revival of chemical dosimetry that now relied on absorption measurements as the analytical method of interpreting the extent of a chemical reaction as a response to radiation (Chorzempa 1971).

In the meantime, the increasing industrial participation in atomic energy programs across the globe, the development of commercial nuclear power, the early nuclear accidents in both research and industrial settings, the health effects of radioactive fallout due to bomb testing especially between 1954 and 1963 (Divine 1978), and the increasing public anxiety over the uses of nuclear power contributed to making health and radiation safety an ever-present public concern (Creager & Rentetzi 2022). The actors involved—state officials, industrialist, politicians, diplomats, scientists, physicians, radiologists, and patients, among others—realized that radiation exposures had already occurred and most probably would occur again in the future, outside of specialized research laboratories. It was because of this realization that radiation safety became a public concern, radiation protection a truly international political matter, and radiation dosimetry a scientific field of key diplomatic and political importance. It was also during this period that the United Nations started to develop an international regulatory system of ionized radiation risks based heavily on the geopolitical division of the world and shaped by Cold War politics. UN related agencies such as the World Health and International Labor Organizations now took the lead. But the crucial event was the establishment of the International Atomic Energy Agency (IAEA) in 1957.

The World Health Organization’s early attempts to plan a working program on radiation protection started in 1953. Two years later, an officer was appointed to the WHO Headquarters to advise the General Director on relevant initiatives concerning the protection of health in persons exposed to risks from ionizing radiation. By 1956 the World Health Assembly recognized radiation protection as a global public health issue and the WHO assumed responsibilities for training workers, collecting relevant information about medical problems related to radiation, as well as cooperating with competent technical bodies on standardization. The same year both the International Commission on Radiological Protection (ICRP) and the International Commission on Radiological Units (ICRU) drafted an official cooperation agreement with the WHO, inviting representatives from the organization as observers to their future conferences (World Health Organization 1958: 289–296; de Chadarevian 2015).

As for the International Labor Organization (ILO), two months before the UN International Conference on the Peaceful Uses of Atomic Energy, which took place in Geneva in 1955, it adopted a resolution emphasizing the need to ensure that atomic energy was going to be used for peaceful purposes, while also drawing attention to the social implications of this development. The ILO had of course a pre-war history in the field, being one of the first organizations to recognize that radiation workers (those working with X‑rays and radium) had a right to compensation in case of occupational injuries stemming from the use of radiation sources (Anonymous 1961). In 1949 a group of experts invited by the ILO had put forward what became the first set of international safety and health standards for the industrial use of X‑rays and radioactive substances. By the time of the Geneva conference, the organization was ready to submit a report that described its own activities in the field of radiation protection (ILO 1955).

Nevertheless, the establishment of the IAEA in 1957 fundamentally changed the entire international regulatory system of radiation protection. It was the only UN agency with statutory responsibilities to draft radiation standards concerning not only its own internal operations but the entire spectrum of the uses of radiation (IAEA Statute, article III, paragraph 6).^2^ Regulation became an instrument of social and political management, a means to drive development in postcolonial states (Abraham 1997) and thus to redefine global inequalities of power. It also proved to be a matter of political dispute among UN agencies, established international disciplinary organizations, state and non-state actors, groups of prominent scientists, and uneasy diplomats. Ever since the establishment of the IAEA, the history of radiation protection has gone beyond national borders and entered a broader context of international relations and diplomatic negotiations where centralized global institutions and science diplomats play significant roles (Kyrtsis & Rentetzi 2021).

This article draws attention to the role of the IAEA in shaping radiation dosimetry practices, instrumentation, and standards in the late 1950s and 1960s. It traces the beginnings of the IAEA’s radiation dose intercomparison program which targeted all member states and involved the WHO so as to standardize dosimetry on a global level. To standardize dosimetric measurement methods, techniques, and instruments, however, one had to devise a method of comparing absorbed dose measurements in one laboratory with those performed in others with high accuracy. In 1964 the IAEA thus started to build up what I call the *global experiment*, an intercomparison of radiation doses with participating laboratories from many of its member states. To carry out the process of worldwide standardization in radiation dosimetry, I argue, an organization with the diplomatic power and global reach of the IAEA was absolutely necessary. Thus, the idea of a *global experiment* indicates a novel understanding of the experimental process. What counts as an experiment is now a process designed and strictly regulated by an international organization, it takes place simultaneously in several laboratories across the globe, while the experimental data is centrally owned and alienated from those that produced it.

Already in the late 1980s Samuel Walker alerted us to the fact that radiation protection in the context of the US Atomic Energy Commission was a deeply social and political issue. Scientific controversies over the risks of fallout or the radiation released by nuclear power plants quickly turned into nasty personal and politicized disputes over radiation risks and public health, involving governmental actors (Walker 1989, 1994, 2000). In producing and implementing radiation standards and developing radiation dosimetry, the UN regulatory system of radiation risks utilized not only politics but state and international diplomacy. The IAEA’s role, which entailed a number of coexistent technical and diplomatic challenges, was especially decisive in this process. In other words, I suggest that multinational scientific-diplomatic gravity did not precede the IAEA’s involvement in standardization but resulted from the very acts of organizing and carrying out the still ongoing *global experiment* in dosimetry.

In what follows, I provide insights into the establishment of the IAEA’s dosimetry laboratory in the early 1960s and the development of the joint IAEA/WHO postal dose comparison service to their member states on the IAEA’s initiative. In the midst of Cold War geopolitical tensions, both of these technoscientific endeavors proved to be political and diplomatically complex. In the absence of dosimetry standards for directly absorbed dose calibrations in radiotherapy and suitable dosimeters for global distribution, the IAEA organized the first trial postal dose comparison for electron beams in 1964–65, with eleven participating institutions and using Fricke dosimeters. The next year more systematic investigations led to a second intercomparison trial, and another year later a third one followed. What had been originally planned as a one-time experiment among several institutions in Europe proved to be an endless experiment involving institutions from around the world and testing the power of diplomacy on several fronts.

By 1967 the IAEA had succeeded in providing its postal dose intercomparison service in collaboration with the WHO. It had also established the foundation of a network of secondary standard dosimetry metrological centers in key regions and on a global level. The *global experiment* in radiation dosimetry—conducted under what were supposed to be predetermined experimental conditions—led to the standardization of dosimetric practices, instruments, and even the architecture of dosimetry laboratories across several member states. Getting the radiation dose correct presupposed diplomatic and political negotiations without precedence in the context of the newly created IAEA, an international organization that hoped to achieve the regulation of nuclear technologies in the medical, research, and industrial sectors worldwide.

## Establishing the IAEA’s Dosimetry Laboratory

On October 15, 1958 a radiation accident took place at the Vinča Nuclear Institute near Belgrade (Higuchi and Hymans 2021; IAEA 1962; Anonymous 1960).^3^ An uncontrolled run of the institute’s heavy-water nuclear reactor resulted in six researchers receiving massive radiation doses. They all developed severe radiation sickness and were rushed to the Boris Kidric Institute for first aid. The next day the patients were all flown to Paris to receive an experimental treatment that was the only available medical option for cases such as theirs: a bone marrow transfusion (BMT) (Kraft 2009). Five of the six patients received infusions of bone marrow while the other one went through conventional treatment with medication and blood transfusions. One of them died one month after the accident, and eventually the rest all rejected the transplants. But it was probably those transplants that contributed to their survival before rejection (Mathé et al. 1959; Ninković 2000; Jansen 2005; Oliveira 1997).

The accident and its aftermath made headlines in major newspapers, including the* New York Times* (Anonymous 1959c). On April 17, 1960, the Yugoslavians announced that the reactor at the Vinča Nuclear Institute was going to be switched on again. The reason was the increasing international interest in reconstructing the accident in order to accurately estimate the radiation doses received by the physicists. This was expected to provide a better understanding of how to resolve issues of radiation protection related to the construction of industrial nuclear reactors and clarify whether the used medical method (BMT) was promising in case of radiation overexposures, especially in industrial settings.

Indeed, to Sterling Cole, the ranking US Congressman who agreed to head the IAEA during its formative years, the reconstruction of the accident was “of major importance to the Agency” because it could inform their safety criteria in the construction and operation of nuclear reactors. Karl Morgan, director of the health physics division at the Oak Ridge National Laboratory, hastened to get involved in the discussion. He confirmed that “in view of the extreme importance of obtaining an accurate estimate of the dose received by these individuals,” the US team was willing to participate in a joint project. “Such data can be extremely useful” continued Morgan, naming the assignment of emergency doses following accidents in the nuclear industry as a major reason.^4^ Eventually, in April 1960, Morgan’s scientific team joined the newly founded IAEA in locally reconstructing the accident (Morgan 1998).

Following this project, the IAEA organized a meeting in Vienna a few months later, inviting leading scientists from 33 countries to discuss issues regarding radiation dosimetry. In his closing speech, Cole emphasized the importance of advancing knowledge in the field of radiation dosimetry and made clear that the Agency would take appropriate action to help meet this need. Very soon it actually did. According to Horst Eisenlohr, later head of the dosimetry section, that same year the IAEA established its own dosimetry laboratory. The physicist Johann Nagl from Vienna’s Technical University was appointed as its head (Eisenlohr 2010).

The idea of adding a research laboratory to a diplomatic agency did not develop as smoothly as one might expect. Already within the IAEA’s Preparatory Commission in 1957 the establishment of a scientific lab had been a controversial issue. As David Fischer writes of the IAEA early days, “hardly any matter could be discussed without provoking lengthy, ideologically tinged, arguments” (Fischer 1997: 77). Fischer served as Assistant Director General for External Affairs throughout the 1980s and later documented the history of the Agency. Referring especially to the establishment of its laboratory, he speaks of the “strong opposition” of the USSR and some Western countries (Fischer 1997: 80).

The man who orchestrated the planning of the laboratory was Henry Seligman, the IAEA’s Deputy Director General for Research and Isotopes during Cole’s administration (1957–1961). Seligman, whom the *New York Times* had characterized as “Britain’s No. 1 isotope salesman” (Anonymous 1955) had been head of the isotope division of the British Atomic Energy Research Establishment in Harwell before joining the IAEA. An enthusiastic supporter of the use of radioisotopes in industry and medicine, Seligman strongly influenced Cole on how to develop the Agency’s laboratory and its radioisotope and dosimetry programs (Sigurbjörnsson 1993). As Otto Suschny, a Viennese chemist who in 1961 became the head of the Agency’s Low Level Radioactivity Laboratory, later remembered,

He [Seligman] alone had the vision to realize that such facilities were essential not only to provide a solid foundation for the unique scientific and technical tasks to be performed by the new organization but also to ensure its support by the scientific community […]. He also had the skill and managerial capability to put his idea into practice in spite of considerable opposition from some colleagues and from several Members of the Board of Governors who did not share his vision, and of formidable practical and financial difficulties. (Suschny 1997: 213)

Indeed, in the final report of the Preparatory Commission in 1957 it was suggested that it could be a “very valuable service” to the Agency’s member states to establish methods of measuring radioactive samples and calibrating measuring equipment. Cole took up the Commission’s recommendations and proposed the topic to the Board of Governors meeting on June 16, 1958. During the critical years 1958 and 1959, heated debates took place in several IAEA board meetings. The major financial investments required for the establishment of the laboratory were just an excuse. Aspects of epistemic power in the Cold War context was the true issue at play. An intense exchange took place during the Agency’s General Conference held on October 4, 1958. Both the Soviet representative Vasily Emelyanov and the Yugoslavian Slobodan Nakicenovic argued that the Agency could use existing laboratory facilities instead of building expensive new ones. Emelyanov went so far as to opine that the Agency’s plans did not raise a “single serious issue”; instead there were irrelevant discussions about setting up a laboratory “intended for minor activities.” And Pavel Winkler from Czechoslovakia warned the General Director that a decision to set up a scientific laboratory might lead to the Agency becoming a fundamental research organization instead of an “organization for broad international cooperation and assistance to under-developed countries” (Winkler 1958).

The representative of the Netherlands, the United Kingdom, France, and in particular the United States insisted that there was no doubt that such a laboratory was critical for the Agency’s work as it could provide several services to member states. Trying to reach a middle ground, Carlos Sánchez del Río, the representative from Spain, suggested caution before any decision was taken. At stake were the IAEA’s power in defining research projects on nuclear science and technology on a global level and the potential of it becoming a center for fundamental research controlled by the US and their close allies. At the end of the day, the conference voted in favor of setting up the laboratory and Cole moved forward with its establishment (Anonymous 1959a: 15). His decision was backed up by President Eisenhower’s generous gesture of donating a mobile radioisotopes training laboratory to the Agency just before the second IAEA General Conference in 1958 (Rentetzi 2021; Mateos & Suárez-Díaz 2015a, b, 2019).

In the Agency’s press release of April 9, 1959, it was confirmed that the laboratory plans had been approved and that the “functional laboratory” was to be built on a site adjoining the Austrian reactor and atomic laboratories at Seibersdorf, near Vienna. Most probably, the decision to place the lab that far from the Agency’s headquarters was influenced by the fact that the new premises were going to use the chemical and low-activity waste disposal system that was already set up by the Austrian “Studiengesellschaft für Atomenergie” (SGEA) and served its own reactor and labs. John McCone, the US Representatives to the IAEA announced the United States’ donation of $600,000 dollars in order to partially cover the costs of constructing and equipping the Agency’s laboratories, while the Netherlands donated the first equipment to be used (Kammerhofer 2011).^5^

The initial idea was to create a laboratory “as flexible as possible” so as to serve the Agency’s statutory responsibilities, which were the regulation and production of radiation standards. But while the new laboratory complex was not planned to be completed before 1961, Cole recommended that the “work could very well start on a small scale in some rooms of the basement of the Grand Hotel which are not required for other purposes” (Kammerhofer 2011: 3). Affirming the “not to lose momentum” kind of argumentation, Suschny later recalled that “[f]acilities for radioactivity measurements and dosimetry were rapidly set up […]. Very soon the basement of the Grand Hotel was bustling with activity.” Taking advantage of pre-existing fume cupboards that served the hotel’s kitchen, the first dosimetry work and the radiochemical laboratory were both set up in that basement along with an electronics workshop (Suschny 1981: 214).

Early laboratory work was centered on a program of absolute radionuclide calibrations, badly needed at the time because of the discrepancies that appeared in measurements carried out in different laboratories around the world. In his recollections, Munir Ahmad Khan, Chairman of the IAEA Board of Governors, conjured up a vivid picture of the “informal and invigorating” atmosphere of those days. At the same time, his observations reflect the unique mingling of nuclear science and diplomacy in action. In the lobby of the hotel, the makeshift IAEA headquarters, one could come across distinguished scientists such as the British Nobel Prize winner in physics in 1951, John Cockroft, or great diplomats such as the Soviet Vyacheslav Molotov. “During lunch hour one could stroll along the Kärntnerstrasse,” Khan recalled, “or go to the cellar to imbibe the pioneering spirit of Dr Henry Seligman’s analytical laboratory” (Khan 1987: 7).

**Fig. 1:**
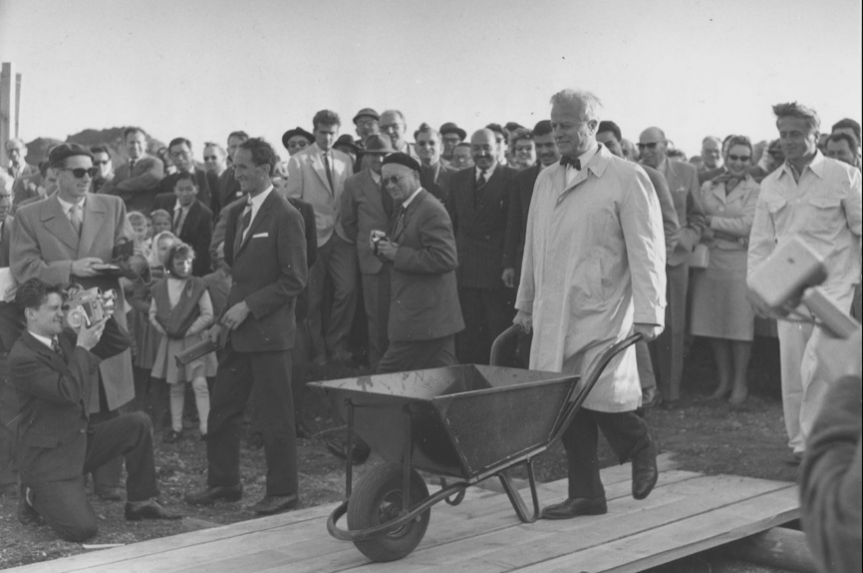
William Sterling Cole, the first Director General of IAEA, pours the first load of concrete into the foundations of the new laboratory to inaugurate construction on September 28, 1959. The laboratory began operating two years later. (Courtesy of the IAEA Archives)

The laboratory remained in the kitchen for two years, until the larger-scale research facilities were constructed in Seibersdorf. In September 1961, two months before Cole left office and was succeeded by Sigvard Eklund as the Agency’s Director General, the Seibersdorf laboratory was finally inaugurated. “The establishment at Seibersdorf is the world’s first full-fledged laboratory of a truly international character,” announced the *IAEA Bulletin* (Anonymous 1962a). All laboratory sections were moved from the Grand Hotel’s kitchen to the new location, except for the tritium and hydrology and the medical sections. The section on metrology and standardization, that on environmental contamination, as well as the chemistry laboratory and the electronics workshop were now operating in Seibersdorf (Kammerhofer 2011: 7). By the time the move was completed in 1961, the Agency had added to its operations a food and agriculture development section. A new allocation of laboratory space along with an extension of the existing space in Seibersdorf were already in demand (Suschny 1981). (Fig. 1).

The laboratory was designed to fulfill five objectives: a) the metrology of radionuclides and the preparation of radioactive standards, b) the calibration and adaptation of measuring equipment, c) the quality control of special materials for nuclear technology, d) measurements and analyses in connection with the Agency’s safeguards and health and safety program, and e) services for member states undertaken by means of the facilities needed for the other activities. In these early years the IAEA invested most of its attention and resources to radiation dosimetry, in particular concerning radiation therapy.

In August 1959 a group of twenty experts from twelve countries jointly convened by the IAEA and the WHO met to review issues related to the use of radiation for the treatment of cancer. Up to that time, malignant tumors had been treated either by using radium applicators, which had been popular especially before World War II, or with high-energy radiation in the form of gamma or X‑rays. The IAEA study group concluded that both high-energy gamma radiation, produced from large sources of radioactive materials like cobalt-60 or cesium-137, and electron beams, produced by high-voltage particle accelerators, were the future of radiotherapy.^6^

It was suggested that the two United Nations organizations should invest in training medical personnel worldwide through their technical assistance programs. Moreover, the determination of radiation doses for clinical practice and the standardization of dosimetry methods were both crucial for the success of the overall program (Anonymous 1959b). The IAEA’s newly established dosimetry lab was tasked with the preparation and testing of a system that would allow the Agency to provide calibration and dose comparison services simply through the mail. It was the first time in the history of radiation that an international political and diplomatic organization was planning to devise a technoscientific system of standardizing dosimetry on a global level. And indeed, soon after, the IAEA embarked on a major dosimetry project—a *global experiment* without precedent—that led to its establishment as the key metrological organization on radiation on the planet.

**Fig. 2:**
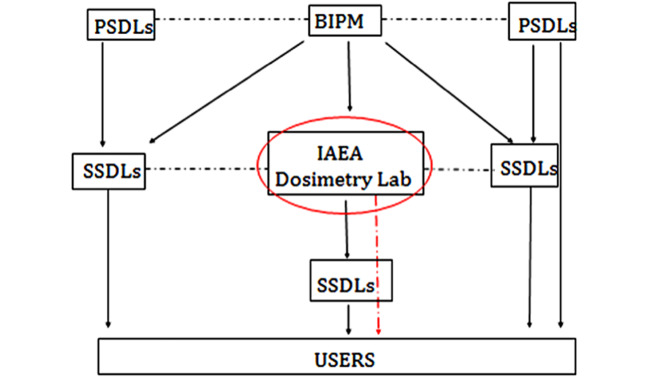
A simplified representation of the global measurement system for radiation dosimetry. The dotted lines represent comparisons of primary and secondary standards and the arrows represent calibrations traceable to primary standards. The red-dashed arrow represents exceptional calibration of a user instrument by the IAEA in the event that a country has no SSDL and limited resources. *BIPM* stands for the Bureau International des Poids et Mesures; *SSDLs* stands for Secondary Standards Dosimetry Laboratories; *PSDLs* stands for Primary Standards Dosimetry Laboratories

Today the IAEA’s Dosimetry Laboratory plays a key role in radiation protection throughout the world, ensuring the maintenance of high standards and guiding the calibration of radiotherapy beams in both low- and high-income countries. It is the central laboratory in the international measurement system for radiation dosimetry, which consists of a network of about 80 Secondary Standard Dosimetry Laboratories (SSDLs) jointly operated by the IAEA and the WHO (Fig. 2). The laboratory calibrates more than 50 ionization chamber systems, verifies some 450 beams from radiotherapy equipment in hospitals across the globe, and provides a follow-up program for hospitals that report dosimetry deviations—including, if needed, on-site visits by IAEA experts and training in radiotherapy equipment.

## Setting Up the First Postal Radiation Dose Intercomparison Experiment

The assistance that the Agency provided to its member states in the medical sector comprised four levels. First, the IAEA encouraged medical clinics in member states to employ physicists in radiotherapy institutions and establish physics services in individual hospitals, thus enforcing the establishment of medical physics as a distinct and strong discipline. Second, it offered specialized training in radiation physics to those wishing to staff their newly established centers. Third, the Agency supported those physicists already employed in the medial sector with available data, advice, and networking opportunities. For example, starting in 1961, the IAEA made a direct financial contribution to the ICRU, the commission responsible for establishing a unit for measurement of radiation when applied in medicine. The three international organizations—ICRU, IAEA, and WHO—also formed an advisory panel on clinical medicine. Finally, at the request of its member states, the Agency offered in its headquarters in Vienna a whole-body counter for the measurement of doses received by radiation workers or overexposed patients (Anonymous 1963: 25; Anonymous 1962b).

This multilevel service demanded reliable radiation dosimetry. And in 1964 the time was finally ripe to provide it. “The number and scope of requests for advice and support in nuclear medicine have substantially increased in 1964,” the IAEA’s Board of Governors realized, fully expecting this trend to continue in 1965–66 (Anonymous 1964: 21). In view of these demands, the Agency initiated a dosimetry project, aiming to access the reliability and accuracy of radiation protection measurements in a small number of European laboratories. This plan was conceived at the Symposium on High-Energy Electrons that took place at Montreux, Switzerland, in September 1964.^7^

During an “informal session,” a small group of physicists—among them Nagl, representing the IAEA’s dosimetry lab—decided to test a simple and relatively inexpensive calibration method. In February 1965 the plan was already in place, and Seligman sent a circular letter to a number of European laboratories describing the project to be undertaken in March and April of that year:

As a first attempt to make a contribution to the improvement of the present situation, the International Atomic Energy Agency plans to distribute four intercomparison chemical dosimeters to institutions interested in the dosimetry of high-energy electron beams. This might be followed by similar intercomparisons of dosimetric measurements involving other types of instruments such as thermoluminescent dosimeters.^8^

Nagl and the physicist Alexandre Sanielevici, who had previously worked at Marie Curie’s lab in Paris and was now the Deputy Director of the IAEA’s Division of Research and Laboratories, took over the task. In order to achieve reliable dosimetry, IAEA scientists had to compare absorbed dose measurements in high-energy electron beams from a given laboratory with those measured at its own laboratory. Based on the results, the next step was to calibrate each laboratory’s equipment according to the IAEA standard. Although the idea was simple, its execution proved to be very demanding since difficult decisions had to be made when it came to both the devices for measuring radiation and to comparing the measurements.

It is important to recall that the history of defining the ways we measure radiation, as well as that of the concept of the dose, has been long and complicated. In the early 1920s the dose was defined in a pharmacological sense as the quantity of radiation given to a patient. This view changed only in the 1940s when scientists finally developed the notion of the absorbed dose, that is, the quantity of radiation energy absorbed per unit mass of the irradiated body. This concept of the absorbed dose was used in the calculation of radiation uptake in living tissues (Wyckoff 1980; Walker 2000). But comparing measurements also required the right tool, called dosimeter. At the time the most reliable seemed to be the Fricke dosimeter, a well-known and widely used dosimeter designed in 1927 by physicists Hugo Fricke and Sterne Morse (Fricke & Morse 1927).

Fricke was a Danish physicist who had earlier worked with Niels Bohr in Copenhagen and subsequently in Lund with Manne Siegbahn. In 1919 he moved to the United States and worked first at Columbia and then at Harvard before he ended up in Cleveland, Ohio, as director of the new biophysical laboratory of the Cleveland Clinic Foundation. In 1925 he had already founded the Victoreen Instrument Company which produced commercially the Victoreen dosimeter, widely used in the nuclear industry. The dosimeter that acquired Fricke’s name was a kind of chemical dosimeter that contained a corrosive liquid (Ferrous sulfate solution) in a “ground-glass stoppered, radiation-resistant, quartz spectrophotometer cell” (Soares et al. 1987: 1). This instrument was for many years the only chemical dosimeter with such high accuracy and precision (Oller et al. 1969).

The principle on which the Fricke dosimeter was based is the fact that ionizing radiation when passing through matter could displace an electron from its orbit around a nucleus. The displaced electron produces further chemical reactions with the medium, leading to new products. When the medium is human tissue, then chemical reactions lead to cell damage and often death. But if the medium is a particular solution in which the reaction products are stable and easily measured, then this solution could be used as a dosimeter. Fricke dosimeters were widely used in radiation biology and medical physics. When irradiated, the Fricke solution produces ferric (iron ions with oxidation number of +3) in proportions that are analogous to the absorbed dose deposited in the solution. The chemical changes that the solution undergoes lead to changes in optical properties that can be measured by simple optical means. Thus, the ferric concentration is measured by absorption spectroscopy, and this is why Fricke dosimeters are placed in spectrophotometer cells.

In view of the above, the IAEA team favored the use of Fricke dosimeters in their first experimental trial. On May 3, 1965, they sent the first batch to ten institutions throughout Europe and to New York’s Memorial Hospital. “The Agency considers to distribute the Fricke solutions only as a first preliminary step, mostly to find out if this technique is useful for the dosimetry of high energy electrons” explained Sanielevici to John Laughlin, director of the Sloan Kettering Institute for Cancer Research in New York.^9^ In total, eight countries were involved in the first trial: Belgium, Finland, France, Germany, Italy, Sweden, Switzerland, and the US. It is important to note that none of these invited institutions was located in the Soviet Union or any of the Eastern Bloc countries that had signed the Agency’s statute. Cold War politics clearly defined participation in the first trial.

**Fig. 3:**
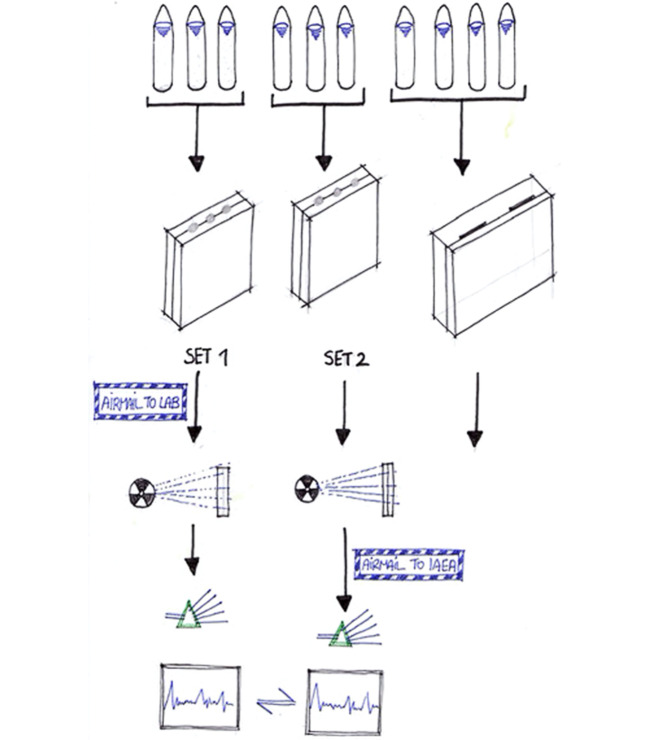
Here the experimental protocol as it was described in a circular letter sent by the IAEA to the eleven participating institutions is shown. (Courtesy of Spiros Flevaris)

The IAEA package contained four Fricke dosimeters—samples A, B, C, and D—sent free of charge to each institution (Fig. 3). The participants were advised not to irradiate samples A and B. Sample A was going to be mailed back to the IAEA and used as reference for checking possible changes in the physical or chemical properties of the solution during the trip. Sample B was evaluated for optical density by the participating institution. The other two samples, C and D, were each placed in a relatively small (6 × 10 × 15 cm) phantom of water-equivalent material. Both of them had to be irradiated under very specific conditions, carefully described by the IAEA team, and with a total dose of 5,000 rad coming from a 30 MeV electron beam. The measurement of this dose was supposed to be done with the institutes’ monitor dosimeters. Participants were advised to send Sample C back to the IAEA dosimetry lab for evaluation. Sample D was going to be evaluated by the participating institution itself. Thus, the institution had to evaluate two samples—B and D—and report the results. A document entitled “Procedure” accompanied the package of dosimeters that was sent to each laboratory, which described in detail the conditions under which it ought to perform the experiment.^10^ When a package left the IAEA headquarters in Vienna, the participating institution was informed by cable about the exact date so as to expect it. The note simply read, “kindly requested to return immediately irradiated Fricke dosimeters.”^11^

The Fricke dosimeters proved to be very precise. Dr. Pierluigi Cova from the radiotherapy department of Casa Di Cura Sant’Ambrogio in Milan explained to Sanielevici: “We usually employ ionization chambers (Victoreen and PTW) for the dosimetry of high-energy electrons and we know that they are not suitable for a correct determination of the real absorbed dose […]. We therefore think that, until a calorimeter [that is] completely tissue equivalent will be realized, the better evaluation of absorbed dose must be made by means of Fricke dosimeters.”^12^

Laughlin, a pioneer of radiation therapy in New York, informed Sanielevici that “we have had under way in the United States a somewhat similar intercomparison program among a number of institutions.”^13^ Laughlin had studied with Donald Kerst, the inventor of the betatron, at the University of Illinois where he also carried out nuclear research on the university’s cyclotron designed by Gerald Kruger and, in 1950, initiated the first therapeutic use of high-energy electrons (Luther 1986). In the early 1960s, Laughlin participated in the joint ICRU/ICRP study group on radiation dosimetry. Following an invitation from the United Nation’s Scientific Committee on the Effects of Atomic Radiation (UNSCEAR), the group ventured to study the radiation doses received by patients during medical treatments with ionizing radiation (Taylor 1979: 9.272–9.274). Hoping to gain from Laughlin’s experience, Sanielevici asked for his participation in the first intercomparison, although his institution was not located in Europe.

On March 2, 1965, Nagl informed all invited institutions that they had received “favorable replies” from the majority of them so that the experiment would soon take place.^14^ Scandinavian institutions were especially eager to participate. They already had an intercomparison program running for the new betatrons in Copenhagen, Oslo, Helsinki, Gothenburg, Lund, Stockholm, Umeå, and Örebro.^15^ Three of the hospitals, those in Gothenburg, Lund, and Umeå, all in Sweden, were invited by the Agency to participate. The first batch of dosimeters was sent out a day later. The Radiation Physics Department of the Royal Medical School of Umeå was impressively fast in performing the experiment and providing data. “We obtained your dosimeters yesterday,” wrote Gunnar Hettinger to Sanielevici, “irradiated and returned them by air the same day.”^16^ Not all institutions were this fast. Wolfgang Pohlit from Frankfurt reported that carrying out of the experiment had been delayed because of necessary repairs to their betatron.^17^ Pohlit was working under the direction of one of the most influential biophysicists of his time, Boris Rajewsky, at the Max Planck Institute for Biophysics.

By June 18, all participating institutions had concluded their measurements and returned their results. The IAEA team circulated a new letter to all institutions including the results and a corrected formula for calculating the absorbed dose which had been wrongly reported in their first letter.^18^ They clearly handled the procedure with great discretion: the final review of the experiment, which is not included in the available archival material, mentioned participant institutions by number without their names, thus making identification impossible, and the list of final results sent out by Sanielevici on October 21 indicated only the number of each institution as well. The IAEA’s discretion and diplomatic phrasing even of scientific data were indeed novel to the experimenters involved. Diplomatic language had already permeated scientific language. As Sanielevici informed all of them, the next intercomparison experiment was already planned for early 1966. He asked for suggestions regarding the details of the planned intercomparison and also for new interested participants.

## The Second Intercomparison Experiment

On December 20, 1965, right after the end of the first intercomparison experiment, Sanielevici contacted Victor Ivanov, the scientific adviser to the resident representative of the Soviet Union to the Agency. He informed him about the experiment, attached a summary, and announced that the IAEA was planning a second intercomparison “aimed at calibrating routine dosimeters for high-energy electron beams used for radiotherapeutical or other purposes.”^19^ His kind request was whether Ivanov could inform the IAEA’s dosimetry group of any institutions in the Soviet Union willing to participate in this second trial. This contact resulted in a dosimetry study tour in the Soviet Union ending in 1971.

With a circular letter sent on January 20, 1966, a number of institutions were invited to participate in the new intercomparison. They were informed that the reproducibility of the results obtained from the first experiment was about +−2%. This second distribution of Fricke dosimeters targeted institutions with accelerators. They wanted to finally compare the radiation dose measured by the Fricke dosimeter that the IAEA provided with the reading of the dosimeter that each institution used routinely in their daily work. The letter listed 13 institutions. An interesting handwritten note (“this letter was also sent to the joining persons”) reveals a slightly less guardedly attitude on the IAEA’s part. The institutions, once again, were located in Europe, in nine different countries, besides Laughlin’s institute in New York.

By the time the IAEA team initiated the intercomparison experiments, Laughlin had already conducted a series of eight intercomparisons between his own Memorial Hospital for Cancer and Allied Diseases and the M.D. Anderson Hospital in Houston (Laughlin et al. 1965). The core of their work was to investigate the stability of irradiated dosimeters during air travel. Samples of Fricke dosimeters in Pyrex glass vessels were sent back and forth between the co-operating institutions. They also used a second kind of dosimeter, stored in polyethylene capsules containing 174 mg of lithium fluoride powder, known as TLDs. The goal was to compare the behavior of both Fricke and TLDs when traveling. As Garrett Holt, Laughlin’s colleague, informed Sanielevici, “we have come to the conclusion that for the accuracies required in practical dosimetry—that is perhaps an accuracy of +−2%—the lithium fluoride dosimeter is the most convenient to use for this purpose.”^20^ The Agency, however, insisted on using Fricke dosimeters in its second intercomparison trial.

On March 29, 1966, Nagl sent the dosimeters to the participant institutions. The ampoules were made of glass with thin walls, filled with ferrous sulfate dosimeter solution, sealed, and placed in a MIX‑D phantom brick, a water-equivalent material. The institutions were asked to irradiate the phantoms with 5,000 rads and return them to the Vienna laboratory for evaluation.^21^ The trial went smoothly and on July 12, Nagl was almost ready to publicize the result. As he admitted to M. J. Day from the Regional Medical Physics Unit of the Newcastle General Hospital, “the results were in very good agreement with the values of institutes which have done some research with this dosimetric system.”^22^ One day later Sanielevici dispatched a number of identical letters to participant institutions to inform them individually about the final results of the experimental trial.

Overall, these first two intercomparison experiments were conducted mainly because the IAEA wanted to “check the feasibility of distributing Fricke dosimeters by air-mail, to irradiate them in various places and to return them by air for evaluation.”^23^ By October 1966 the Agency scaled up its project. It decided to offer this kind of intercomparison service to interested institutions in member states once a year. As Nagl explained to the Institute for Radiation Technology in Karlsruhe, the ICRU was planning to propose the Fricke dosimeter as reference standard and the IAEA had decided to harmonize its service with them. But when Tran Hoang Hai from the Department of Betatron in Lausanne asked Nagl whether they “can afford comparison and standardization of dosimeters in different laboratories with your Standard in IAEA laboratory,” Nagl was very careful not to claim standardization as the Agency’s actual project.^24^ He argued:

These measurements are comparison measurements. We avoid the expression “standardization” because until today the ICRU has not recommended a primary standard for dosimetry of high-energy radiation. Therefore, we do not use “standardization” in spite of the fact that the ferrous sulphate dosimeter is a recommended secondary standard.^25^

To understand the Agency’s caution, one has to view it in the context of international developments in preparing and negotiating radiation standards. The IAEA was determined to create a niche for itself among regulatory institutions with a long and strong tradition in radiation protection. Given the IAEA’s powerful political position, the others had to strive for a compromise. What seems to have been decisive was the personal acquaintance of Lauriston Taylor and Sterling Cole. Taylor, the American pioneer in radiation protection and chairman of the ICRU at the time, knew Cole from his time as a member of the Joint Committee on Atomic Energy in the US. This personal link helped settle the division of labor among the three international organizations in 1960—two of them (ICRU and ICRP) scientific and the third (IAEA) political and diplomatic. As a compromise Cole promised an annual budget to both the ICRU and ICRP. The IAEA was going to produce codes for radiation protection but the philosophy was going to be dictated by the other two organizations (Boudia 2007; Vetter 1966; Taylor 1979: 9.259).

## Interlaboratory Comparisons for Cobalt-60

As the IAEA was taking up a global regulatory role in radiation dosimetry, it was in need of dosimeters that could be easily distributed on a global scale and reach the member states on demand. To advance its work, in March 1967 the Agency planned another interlaboratory comparison, this time in order to check the feasibility of Fricke dosimeters for cobalt-60 teletherapy units of gamma rays instead of accelerators with high-energy electrons.^26^ Once again, the Vienna group promised to send a set of ampoules filled with ferrous sulfate solution placed in a MIX‑D phantom block. The participating laboratories were asked to irradiate the ampoules in such a way that the central one receives a dose of 5,000 rad. The monitors routinely used in each laboratory were to record the measurement. The dosimeters then ought to be returned to Vienna by airfreight.

As in previous experiments, Sanielevici involved Laughlin’s group in New York. But Holt, Laughlin’s colleague, raised an issue: why not use TLD dosimeters in the Agency’s trial? As he assured Nagl, “We have had excellent agreement of measurements using lithium fluoride dosimeters [TLDs], but our results with ferrous sulphate [Fricke dosimeters] have not been as good.”^27^ However, the IAEA group was clearly not interested in film dosimetry but mainly in calorimetry, Fricke dosimetry, and ionization chambers. TLDs were not yet in their programmatic plans. In the meantime, the National Bureau of Standards had expressed its willingness to also provide an intercomparison service for electron beams. The dosimeters they planned to use were Fricke as well. The American Association of Physicists in Medicine (AAPM) had already endorsed this plan with its sub-committee on radiation dosimetry being heavily involved in the process. Although Laughlin’s and Holt’s pioneering team in nuclear medicine was pushing for TLDs, then, all of the major regulatory institutions were still favoring Fricke dosimeters.

During the experimental trial that involved cobalt-60 teletherapy units, participant laboratories made clear their requests. “We would certainly like to standardize our dosimeters,” admitted J. F. Diehl from the Institute for Radiation Technology in Karlsruhe to Sanielevici. Others expressed “an ardent desire” to participate, were “sehr interessiert” and “looking forward to this participation.”^28^ This time, besides Europe, participant institutions were located in New Zealand, India, Taiwan, Sudan, and Japan, to which were added several laboratories in the US. Eventually, the 1967 IAEA Laboratory Activities Report stated:

The aim of the work in Radiation Dosimetry is to establish, on an international scale, agreed standards for dosimetry so that the work of different scientists, doctors, laboratories and hospitals in the Member States may be fully intercomparable, and to this extent, more scientifically meaningful. (IAEA 1967: 8)

Indeed, within seven years (1960–1967) the IAEA had succeeded in creating, in collaboration with the WHO, a “modern” radiation metrology system, and had become the key metrological center for radiation dosimetry. Throughout 1967 the demand for the postal dose intercomparison service was such that the Agency decided to scale up and establish a dosimetry section. Its task was “to advise Member States in the use of established techniques and procedures for the measurement of ionizing radiations and the calibration of dosimeters” (Eisenlohr 1983: 16). Focusing mainly on developing countries in Latin America and the Far East in the following years, the IAEA designed and funded regional dosimetry centers. By 1975 there were seven such centers: in Argentina, Iran, Mexico, Nigeria, Romania, Singapore, and Thailand. In 1982 the number of dosimetry centers worldwide had gone up to twenty-three and the funds spent on this operation amounted to more than 1.5 million dollars (Eisenlohr 1985, Eisenlohr 1989).

## Unruly Labs, Unstable Materials, and Uncooperative Airlines

Throughout the early postal dose intercomparison trials, reports and official documents published by the IAEA were, as expected, written in a neutral, strictly technical manner. Take for example Nagl’s and Sanielevici’s article in *Strahlentherapie* published in 1967 as a response to many requests for a detailed description of their experimental procedures. Giving scant attention to the long list of complexities and failures, the many repairs and adjustments that had to be done, or the delays that many laboratories faced, the IAEA group appeared confident that scientific work would proceed more easily as soon as standards were agreed upon. “This dosimetry intercomparison is inexpensive,” they wrote, further asserting that “only manpower and transportation costs have to be taken into consideration” (Nagl & Sanielevici 1967: 566; Nagl et al. 1964).

In reality, while Fricke dosimeters were very accurate, they were extremely laborious to produce, and the instability of materials became obvious in each step of their production. Particular care had to be taken during the preparation, handling, and evaluation of the procedure. Given that organic impurities could affect the final measurements, all glassware used had to be cleaned with chemicals and rinsed several times with distilled water. Spectrophotometer cells also had to be rinsed with distilled water several times throughout the production process. After the procedure was finished, all laboratory utensils had to be carefully stored in dust-free and specially designed cabinets. Preparing the Fricke solution, filling the spectrophotometer cells, and pre-irradiating the first batch of dosimeters for control check usually took a whole day. The personnel had to be especially cautious and wear rubber aprons and face shields (Soares et al. 1987).

And the problems did not end with the production of the Fricke dosimeters. Given that they were very delicate—after all they were thin glass ampoules full of a corrosive liquid—it was generally difficult to handle them. During the first experimental trial, Ulla-Brita Nordberg, a physicist at the Radiophysics Central Laboratory in Lund, informed Sanielevici that “the ampoule no. 78 was broken during the attempt to take it out from the phantom. The solution then was impured.”^29^ Yet, the most troubling part of the whole experiment involved finding a suitable system for mailing the Fricke dosimeters to the participating institutions. “By this test with a small number of Institutes,” Nagl explained, “we would like to try to get some knowledge on the possibilities of mailed Fricke dosimeters.”^30^ Indeed, since they contained a corrosive liquid in unsealed containers, it was complicated to send these dosimeters by mail. In case of air transportation, the Agency had to deal with package regulations, given that the package had to travel in the pressurized compartment of the airplane.

When, for example, Laughlin’s group tried to return the irradiated dosimeters to Vienna for a readout, “the package was delayed at the air express office in New York City for about three weeks before it was finally dispatched to Vienna.”^31^ The long delay between the exposure of the dosimeter and the reading of the density might have had effects on the accuracy of the determination of the dose—but Nagl hastened to assure them that that effect was negligible.^32^ Karl Johan Vikterlöf from Örebro in Sweden expressed similar concerns: “The only small problem I see is that we have no direct air-communication here to Orebro, which means that we perhaps will be delayed some few days due to the necessity of using a combination of air-transport and train-transport from Stockholm to Orebro.”^33^

The “mailing techniques” came up again and again as the major concern of all those involved in the trials.^34^ When the Argonne National Laboratory returned to the Agency the set of their irradiated dosimeters, they included also a copy of their shipping order and detailed info on the route of the package to make sure that it would be received. References to long delays appear in a number of other responses coming from US laboratories. During the third trial the participant laboratory at the University of Chicago did not even receive the sent IAEA package.^35^ But if sending and receiving dosimeters posed a problem in the US, it was an even bigger challenge to clear IAEA packages at the customs in Khartoum, Sudan. “Unfortunately, it has taken a long time to clear these items from the customs here, and we are now waiting for an export permit to allow them to be returned to you” wrote T. J. Davy, an IAEA expert who was sent to Sudan to oversee the establishment of a radiotherapy center in Khartoum, the first of its kind in an African country.^36^

On top of these issues, several airlines refused to transport the Fricke dosimeters on passenger aircrafts since they contained a corrosive liquid and were thus tagged as a “restricted article.” Nagl clearly made an effort to persuade airlines to change their restrictions. As an international organization the IAEA had the power to bring the issue directly to the International Air Transport Association (IATA). On March 22, 1967, Nagl addressed the Association hoping that they could add a new article in the next edition of its restricted articles regulations:

This liquid is in fact a 2% solution of sulfuric acid, has as a matter of fact a lower acid concentration than vinegar and is, according to information of the University of Vienna, so harmless that it could be swallowed. We usually dispatch in one package less than 50 cm3 of this solution in 10 sealed ampoules, which are placed in wax blocks and surrounded by absorbing material.^37^

Adrianus Groenewege, the Association’s secretary, responded within a week. He had already circulated Nagl’s letter to the members of the IATA’s relevant working group and suggested that this matter had to be reviewed at the Association’s annual meeting. Commenting directly on Nagl’s assurance that the solution was totally harmless, Groenewege mentioned that “personally I am rather doubtful about this statement, and I certainly would not suggest to anybody that they attempt to do so [swallow the solution].” On July 26, Groenewege informed Nagl that the IATA working group was persuaded that the “article,” meaning Fricke dosimeters, “does not meet the definition of a corrosive liquid.” Yet it was not considered as completely harmless and thus IATA placed it under “Other restricted articles,” which required special packaging. Nagl thanked him, expressing his hope that this will help to overcome difficulties in the future.^38^

But even if airlines could be disciplined and materials stabilized, and the Fricke dosimeters reached their destinations, the IAEA group had to deal with the unruliness of the laboratory throughout all trials. During the first experiment and after the IAEA announced the results, Garrett Holt, Laughlin’s colleague at Sloan Kettering, reported that the IAEA group had gotten wrong two things for their own case. First of all, for the irradiation of the first batch of dosimeters Laughlin and Holt used a source of 30 MeV and not 5,000 rad as the IAEA team expected. Second, the results of the samples that appeared on the IAEA’s final report did not agree with those reported by Laughlin and Holt. Again, they were not the only ones to point to similar issues. Almost all participant institutions made mistakes of all sorts during the procedure.^39^

In the end, what the IAEA had hoped to be a one-off experiment followed by a routine black-boxed service to its member states, turned out to be an endless experiment that required strictly controlled spaces, materials, bureaucracies, and even architectures. In 1967 the Agency finally also tested TLD dosimeters, which proved much better suited for travel. A year later, at the request of the IAEA, a panel of experts together with representatives from the WHO met in Caracas to discuss dosimetry in radiotherapy. The selection of Venezuela was not accidental. At the time there was not a single laboratory in Latin America that could calibrate dosimeters, and there were only five qualified hospital physicists in the whole region. It took the IAEA and WHO another six years to agree in setting up a global metrological system in dosimetry through the SSDL network.^40^

## Conclusion

Despite the highly sophisticated medical technologies used in radiation therapy, cancer patients around the world continue to receive the wrong radiation doses during their personalized treatments. Linear accelerators inexplicably allow radiation to spill in wider areas than planned, or radiation beams deliver higher radiation doses than is acceptable because of wrong calibration. All this has reinforced the need for getting the radiation dose correct. But, as the historian of biology John Beatty has suggested, this is not a simple, “purely scientific” task. Determining the maximum acceptable dose of radiation is “a matter of social and political importance,” a process of constant negotiations, “masked disagreements,” and consensus (Beatty 2006: 52). What this article suggests is that getting the radiation dose correct has also been a complex *diplomatic* issue. The focus has been on the first global dosimetry experiment performed by the IAEA in the 1960s.

The endeavor was conceived as a well-designed experiment and the IAEA group described not only the exact conditions of the experimental procedure, its geometry, and the materials and instruments needed for the performance. It also provided the right tool for the job, the Fricke dosimeter. However, Nagl, Sanielevici et al. were not the first to do so. The subcommittee on radiation dosimetry of the American Association of Physicists in Medicine (AAPM), of which John Laughlin was a chairman, had already performed similar trials within the United States. Their aim had been to “obtain uniformity of dosimetry,” in short: to standardize dosimetric methods and tools among major US institutions. Fricke dosimeters were suggested as suitable for this purpose. Trying to satisfy a pressing demand, the AAPM also suggested the establishment of a national calibration service (SCRAD 1966: 506).

But while Laughlin’s team transported dosimeters by hand and in open quartz cuvettes, the IAEA’s dosimetry group was obliged to use air transportation. The act of scaling up from a national calibration service to a network of secondary standard dosimetry centers in different regions across the globe demanded a somewhat grandiose project. The Agency’s intercomparison trials developed into *global experiments* that had to be readjusted in the face of unruly laboratories, unstable materials, unreliable transportation services, and uncontrollable airlines. Only an international organization with great political and diplomatic power such as the IAEA was in a position to design, plan, and execute such an ambitious project. When the IAEA finally established a postal dosimetry intercomparison service, still one of the most important services provided to its member states today, and an extended network of SSDLs, it had succeeded in simultaneously imposing its methods, experimental culture, and dosimetry standards on a worldwide level.

## Footnotes

1 “Radioisotopes for Therapy”, *IAEA Press Release* 61/33, 24 May 1961, IAEA Archives.

2 The IAEA Statute is available at https://www.iaea.org/about/statute.

3 See also Slobodan Nakicenovic to Sterling Cole, 8 February 1959; Hubert Lefebvre de Laboulaye to Paul Foster, 29 July 1959, all in SC/645‑1, Health Safety and Waste Disposal-Reactor Safety-Reactor Accidents-Yugoslavia-Vinča Dosimetry Experiment, IAEA Archives (hereafter SC/645‑1, IAEA.)

4 Sterling Cole to Nakicenovic, 23 February 1959, SC/645‑1; Karl Morgan to Danica Kalic and Brainslav Penditch, 12 June 1959, SC/645‑1, IAEA Archives.

5 “IAEA Sets Up Own Laboratory,” *IAEA Press Release* 59/27, 1959, IAEA Archives.

6 See for example Almond (1975); Kraft (2006); Kevles (1997); Feld & De Roo (2003); Kutcher (2009); Leopold (2009); Mallard (2003)

7 Johann Nagl to Wolfgang Pohlit, Max Planck Institute, 1 February 1965, LAB/222‑1, IAEA Archives.

8 Henry Seligman to Werner Schumacher, Röntgen- und Strahleninstitut Berlin, 1 February 1965, LAB/222‑1. IAEA Archives.

9 The participant institutions were the Casa di Cura Sant’Ambrogio in Milan, the Radiophysics Laboratory at Sahlgren’s Hospital in Gothenburg, the Radiophysika Institutionen at the University of Lund, the Institut Jules Bortet in Brussels, the Department of Radiophysics of the Rudolf Virchow Hospital in Berlin, the Radiation Physics Department of the Royal Medical School of Umeå, the Aktiengesellschaft Brown, Boveri & Cie in Baden, Switzerland, and the Max Planck Institute for Biophysics in Frankfurt. See Sanielevici to John Laughlin, Division of Biophysics, Sloane Kettering Institute for Cancer Research, New York, 2 April 1965, LAB/222‑1, Part I, IAEA Archives.

10 Sanielevici to Laughlin, “Procedure”, 29 April 1965, LAB 222‑I, IAEA Archives.

11 Cable note, 20 May 1965, LAB/222‑1, Part I, IAEA Archives.

12 Pierluigi Cova to Sanielevici, 17 February 1965, LAB/222‑1. IAEA Archives. Similar concerns appear also in the letter of Dietrich Frost to Sanielevici, 24 February 1965, LAB/222‑1, Part I, IAEA Archives.

13 Laughlin to Sanielevici, 1 March 1965, LAB/222‑1, Part I, IAEA Archives.

14 See for example Sanielevici to Kurt Linde, Radiophysika Institutionen, Lund, 2 March 1965; Sanielevici to Sampert, 2 March 1965; Sanielevici to Binks, 2 March 1965; Sanielevici to J. Dutreix, 2 March 1965; all in LAB/222‑1, Part I, IAEA Archives.

15 Linde to Sanielevici, 19 February 1965, LAB/222‑1, Part I, IAEA Archives.

16 Gunnar Hettinger to Sanielevici, 7 May 1965, LAB 222‑I, IAEA Archives.

17 Rajewsky to Sanielevici, 17 May 1965, LAB/222‑1, Part I, IAEA Archives.

18 Sanielevici, Circular letter, 18 June 1965, LAB/222‑1 Part I, IAEA Archives.

19 The summary is not part of the archival material that I was allowed to see. Sanielevici to Victor Ivanov, 20 December 1965, LAB/222‑1, Part I, IAEA Archives.

20 Garrett Holt to Sanielevici, 26 January 1966, LAB/222‑1, Part I, IAEA Archives.

21 Nagl to Tran Hoang Hai, Department of Betatron and Gammatron, Hôpital Cantonal, Lausanne, 20 October 1966, LAB/222‑1, Part I, IAEA Archives.

22 Nagl to M. J. Day, 12 July 1966, LAB/222‑1, Part I, IAEA Archives.

23 Sanielevici to Charles Lescrenier, Marquette University School of Medicine, Wisconsin, attached “Fricke-Test Intercomparison,” November 17, 1966, LAB/222‑1, Part I, IAEA Archives.

24 Nagl to Hai, 20 October 1966, LAB/222‑1, Part I, IAEA Archives; Nagl to the Institute for Radiation Technology, 27 October 1966, LAB/222‑1, Part I; Hai to Nagl 12 October 1966, LAB/222‑1, Part I, IAEA Archives.

25 Nagl to Hai, 20 October 1966, LAB/222‑1, Part I, IAEA Archives.

26 Sanielevici, circular letter, 7 February 1966, LAB/222‑1, Part II, IAEA Archives; Sanielevici to Lescrenier, 17 November 1966, LAB/222‑1, Part II, IAEA Archives.

27 Holt to Nagl, 15 February 1967, LAB/222‑1, Part II, IAEA Archives.

28 J. F. Diehl to Sanielevici, 16 February 1967, LAB/222‑1, Part II, IAEA Archives. See also for example Hideo Matsuzawa, National Institute of Radiological Sciences, Japan to Sanielevici, 17 February 1967, LAB/222‑1, Part II, IAEA Archives; J. Becker, University Radiation Clinic, University of Heidelberg to Nagl, 24 February 1967, LAB/222‑1, Part II, IAEA Archives; Melvin Griem, Chicago Tumor Institute, University of Chicago to Sanielevici, 25 February 1967, LAB/222‑1, Part II, IAEA Archives.

29 Ulla-Brita Nordberg to Sanielevici, 24 June 1965, LAB /222‑I, IAEA Archives.

30 Nagl to Laughlin, 2 February 1965, LAB/222‑I

31 Holt to Nagl, 26 January 1966, LAB/222‑1, Part I, IAEA Archives. See also references to these issues in Holt to Sanielevici, 9 February 1966, LAB/222‑I, IAEA Archives.

32 Nagl to Holt, 15 February 1966, LAB/222‑1, Part I, IAEA Archives.

33 Karl Johan Vikterlöf to Sanielevici, 17 February 1967, LAB/222‑1, Part II, IAEA Archives.

34 See for example Alan Jennings, Division of Radiation Science, National Physical Laboratory, Middlesex to Sanielevici, 20 February 1967, LAB/222‑1, Part II, IAEA Archives.

35 L. D. Jefferson, Materials Handling Department, National Argonne Laboratory, to Sanielevici, 17 April 1967, LAB/222‑1, Part II, IAEA Archives; Sanielevici to L. M. Griem, University of Chicago, 18 May 1967; Sanielevici to Lescrenier, 18 May 1967, LAB/222‑1, Part II, IAEA Archives.

36 T. J. Davy to E. Lloyd, IAEA officer for Africa, 24 April 1969, LAB/222‑1, Part II, IAEA Archives; Davy to Nagl, 24 March 1969, LAB/222‑1, Part II, IAEA Archives. See also GC(60)/OR.2, 26 September 2016, 16. IAEA Archives.

37 Nagl to Adrianus Groenewege, Secretary, International Air Transport Association, 22 March 1967, LAB/222‑1, IAEA Archives.

38 Groenewege to Nagl, 30 March 1967, LAB/222‑1, IAEA Archives; Groenewege to Nagl, 26 July 1967, LAB/222‑1, IAEA Archives; Nagl to Groenewege, 7 August 1967, LAB/222‑1, IAEA Archives.

39 Nordberg to Sanielevici, 24 June 1965, LAB/222‑I, IAEA Archives; Frank Williamson, Argonne National Laboratory to Sanielevici, 13 April 1967, LAB/222‑1 Part I, IAEA Archives; Sanielevici, Circular letter, 18 June 1965, LAB/222‑1 Part I, IAEA Archives.

40 The IAEA/WHO Network of Secondary Standard Dosimetry Laboratories, IAEA/WHO/SSDL-99, SSDL Network Charter, IAEA, 1999.

## References

[CR1] Abraham I (1997). Science and secrecy in making of postcolonial state. Economic and Political Weekly.

[CR2] Almond P (1975). Some applications of particle accelerators to cancer research and treatment. Physics Report.

[CR3] Anonymous (1955). Britain Exploits Atomic Waste.

[CR4] Anonymous (1959). Highlights of the 2nd Session of the General Conference. IAEA Bulletin.

[CR5] Anonymous (1959). High Energy Radiation in Cancer Treatment. IAEA Bulletin.

[CR6] Anonymous (1959). Atomic Accident to Be Duplicated.

[CR7] Anonymous (1960). A Unique Experiment: Measurement of Radiation Doses in Vinca. IAEA Bulletin.

[CR8] Anonymous (1961). ILO and Atomic Energy. IAEA Bulletin.

[CR9] Anonymous (1962). Work Begins at Seibersdorf Laboratory. IAEA Bulletin.

[CR10] Anonymous (1962). Work at the IAEA Laboratory. IAEA Bulletin.

[CR11] Anonymous (1963). Medical Physics: The Agency’s Contribution. IAEA Bulletin.

[CR12] Anonymous (1964). The Agency’s Programme for 1965–66. IAEA Bulletin.

[CR13] Beatty J (2006). Masking Disagreements Among Experts. Episteme: A Journal of Social Epistemology.

[CR14] Boone MLM, Lawrence JH, Connor WG, Morgado R, Hicks JA, Brown RC (1977). Introduction to the Use of Protons and Heavy Ions in Radiation Therapy. Historical Perspective. International Journal Radiation Oncology, Biology, Physics.

[CR15] Boudia S (2007). Global Regulation. Controlling and Accepting Radioactivity Risk. History and Technology.

[CR16] Chorzempa, Sister Mary Andre 1971. *Ionizing Radiation and Its. Chemical Effects: A Historical Study of Chemical Dosimetry (1902*–*1962).* PhD Thesis, Oregon States University

[CR17] Creager A, Rentetzi M, Bensaude-Vincent B, Boudia S, Sato K (2022). Sharing the “Safe” Atom? The International Atomic Energy Agency and Nuclear Regulation through Standardisation 1. Living in a Nuclear World: Order, Knowledge, and Normalization.

[CR18] De Chadarevian S (2015). Human Population Studies and the World Health Organization. Dynamis.

[CR19] Divine R (1978). Blowing on the Wind. The Nuclear Test Ban Debate 1954–1960.

[CR21] Eisenlohr H (1983). A Modern Approach to Radiation Metrology. IAEA Bulletin.

[CR22] Eisenlohr H (1985). The IAEA/WHO Network of Secondary Standard Dosimetry Laboratories. A Novel Approach to Modern Radiation Metrology. Medical Physics World.

[CR23] Eisenlohr H (1989). Standardizing Radiation Doses in Medicine and Industry. IAEA Yearbook 1989.

[CR24] Eisenlohr H (2010). The IAEA/WHO TLD Postal Dose Audit Service. From 1966 to 2010. SSDL Newsletter.

[CR25] Feld M, De Roo M (2003). History of Nuclear Medicine in Europe.

[CR26] Fischer D (1997). History of the International Atomic Energy Agency: The First Forty Years.

[CR27] Fricke H, Morse S (1927). The Chemical Action of Roentgen Rays on Dilute Ferrosulphate Solutions as a Measure of Dose. American Journal of Roentgenology and Radium Therapy.

[CR28] Higuchi T, Hymans Jacques EC (2021). Materialized Internationalism. How the IAEA Made the Vinča Dosimetry Experiment, and How the Experiment Made IAEA. Centaurus.

[CR29] International Atomic Energy Agency (1962). The Vinča Dosimetry Experiment.

[CR30] International Atomic Energy Agency (1967). IAEA Laboratory Activities. Fourth Report.

[CR31] International Labour Organization (1955). The Protection of Workers Against Ionising Radiations. Report Submitted to the International Conference on the Peaceful Uses of Atomic Energy.

[CR32] Jansen J (2005). The First Successful Allogeneic Bone-Marrow Transplant: Georges Mathé. Transfusion Medicine Reviews.

[CR33] Kammerhofer L (2011). 1961 Seibersdorf-Monaco-Salzburg.

[CR34] Kevles B (1997). Naked to the Bone. Medical Imaging in the Twentieth Century.

[CR35] Khan MA (1987). 1957–1987: Development Through Global Co-operation. IAEA Bulletin.

[CR36] Kraft A (2006). Between Medicine and Industry. Medical Physics and the Rise of the Radioisotope 1945–1965. Contemporary British History.

[CR71] Kraft Alison (2009). Manhattan Transfer: Lethal Radiation, Bone Marrow Transplantation, and the Birth of Stem Cell Biology ca. 1942–1961. Historical Studies in the Natural Sciences.

[CR37] Kutcher G (2009). Contested Medicine: Cancer Research and the Military.

[CR38] Kyrtsis A, Rentetzi M (2021). From Lobbyists to Backstage Diplomats. How Insurers in the Field of Third-Party Liability Shaped Nuclear Diplomacy. History and Technology.

[CR39] Laughlin J (1965). A Report on the Intercomparison by Mailed Dosemeters of High Energy Electron Beams. Physics in Medicine and Biology.

[CR40] Lederman M (1981). The Early History of Radiotherapy. 1895–1939. International Journal of Radiation Oncology—Biology—Physics.

[CR41] Leopold E (2009). Under the Radar. Cancer and the Cold War Critical Issues in Health and Medicine.

[CR42] Luther B (1986). John Laughlin. The Fifty-First Janeway Lecturer. American Journal of Clinical Oncology.

[CR43] Mallard JR (2003). The Evolution of Medical Imaging. From Geiger Counters to MRI. Perspectives in Biology & Medicine.

[CR44] Mateos G, Suárez-Díaz E (2015). Clouds, Airplanes, Trucks and People. Carrying Radioisotopes to and across Mexico. Dynamis.

[CR45] Mateos G, Suárez-Díaz E (2015). Radioisótopos itinerantes en América Latina. Una historia de ciencia por tierra y por mar.

[CR46] Mateos G, Suárez-Díaz E, Krige J (2019). Technical Assistance in Movement: Nuclear Knowledge Crosses Latina American Borders. How Knowledge Moves. Writing the Transnational History of Science and Technology.

[CR47] Mathé G (1959). Transfusions and Grafts of Homologous Bone Marrow in Humans after Accidental High Dosage Irradiation. Revue Française d’Etudes Cliniques et Biologiques.

[CR48] Morgan K (1998). History of the International Radiation Protection Association. Health Physics.

[CR49] Mould R (1993). A Century of X-rays and Radioactivity in Medicine.

[CR50] Nagl J, Sanielevici A (1967). Dosisvergleichsmessungen für hochenergetische Elektronen mit Eisensulfat-Dosimeter. Strahlentherapie.

[CR51] Nagl J, Sanielevici A, Widerøe R (1964). Calorimetric Dose Measurements with 35-MeV Betatron Electron Radiation. Nature.

[CR52] Ninković, Marko M. 2000. Radiation Protection Experience in Yugoslavia. From the Vinča Accident to Nowadays. https://inis.iaea.org/collection/NCLCollectionStore/_Public/39/057/39057192.pdf. Accessed 4 Mar 2022.

[CR53] Oliveira, Alexandre R. 1997. Posthumous Tribute to Professor Henri Jammet—A Brief Biography. REMPAN 97:16–23. http://cidbimena.desastres.hn/pdf/eng/doc12327/doc12327-contenido.pdf. Accessed 4 Mar 2022.

[CR54] Oller WL, Menker DF, Dauer M (1969). Evaluation of the Fricke Dosimeter with Other Dosimetry Systems. Health Physics.

[CR55] Rentetzi M (2021). With Strings Attached: Gift-Giving to the International Atomic Energy Agency and US Foreign Policy. Endeavor.

[CR56] Rentetzi M (2022). Seduced by radium: how industry transformed science in the American marketplace.

[CR57] Sigurbjörnsson B (1993). Professor Henry Seligman in Memoriam. Applied Radiation Isotopes.

[CR58] Soares C, Bright E, Ehrlich M (1987). Fricke Dosimetry in High-Energy Electron Beams NBS Measurement Services.

[CR59] Sub-Committee on Radiation Dosimetry (SCRAD) (1966). Protocol for the Dosimetry of High Energy Electrons. Physics in Medicine Biology.

[CR60] Suschny O (1981). Twenty Years of an International Nuclear Laboratory. IAEA Bulletin.

[CR61] Suschny O (1997). The Agency’s Laboratories at Seibersdorf and Vienna. International Atomic Energy Agency. Personal Recollections.

[CR62] Taylor L (1979). Organization for Radiation Protection. The Operations of the ICRP and NCRP 1928–1974.

[CR63] Vetter H (1966). Nuclear Medicine. A New Discipline. IAEA Bulletin.

[CR65] Walker SJ (1989). The Controversy over Radiation Protection. A Historical Overview. Journal of the American Medical Association.

[CR66] Walker SJ (1994). The Atomic Energy Commission and the Politics of Radiation Protection, 1967–1971. Isis.

[CR64] Walker SJ (2000). Permissible Dose. A History of Radiation Protection in the Twentieth Century.

[CR67] Winkler P (1958). The Board of Governors. IAEA Bulletin.

[CR68] Womack J (2020). Radiation Evangelists. Technology, Therapy, and Uncertainty at the Turn of the Century.

[CR69] World Health Organization (1958). The First Ten years of the World Health Organization.

[CR70] Wyckoff HO (1980). From “Quantity of Radiation” and “Dose” to “Exposure” and “Absorbed Dose”—An Historical Review.

